# The Statistical Scale Effect as a Source of Positive Genetic Correlation Between Mean and Variability: A Simulation Study

**DOI:** 10.1534/g3.119.400497

**Published:** 2019-07-18

**Authors:** Adile Tatliyer, Isabel Cervantes, Nora Formoso-Rafferty, Juan Pablo Gutiérrez

**Affiliations:** * Department of Animal Science, Faculty of Agriculture, Kahramanmaras Sutcu Imam University, Avsar Campus, 46100, Onikisubat, Kahramanmaras, Turkey and; † Department of Animal Production, Faculty of Veterinary, Complutense University of Madrid, Avda. Puerta de Hierro s/n, E-28040-Madrid, Spain

**Keywords:** heteroscedastic model, environmental variability, scale effect

## Abstract

The selection objective for animal production is the highest income with the lowest production cost, while ensuring the highest animal welfare. A selection experiment for environmental variability of birth weight in mice showed a correlated response in the mean after 20 generations starting from a crossed panmictic population. The relationship between the birth weight and its environmental variability explained the correlated response. The scale effect represents a potential cause of this correlation. The relationship between the mean and the variability implies: the higher the mean, the higher the variability. The study was to quantify by simulation the genetic correlation between a trait and its environmental variability. This can be attributable to the scale effect in a range of coefficients of variation and heritabilities between 0.05 and 0.50. The resulting genetic correlation ranged from 0.1335 to 0.7021 being the highest for the highest heritability and the lowest CV. The scale effect for a trait with heritability between 0.25 and 0.35 and CV between 0.15 and 0.25 generated a genetic correlation between 0.43 and 0.57. The genetic coefficient of variation (GCV) affecting residual variability was modulated by the strength reducing the impact of the scale effect. GCV ranged from 0.0050 to 1.4984. The strength of the scale effect might be in the range between 0 and 1. The scale effect would explain many reported genetic correlation and the additive genetic variance for the variability. This is relevant when increasing the mean of a trait jointly with the reduction of its variability.

Currently, interest is increasing in the genetic control of environmental variability for application in livestock breeds. Environmental variability refers to the variation in phenotypes that cannot be explained by genetic variation or identifiable genetic differences (Hill and Mulder 2010). The importance of genetic control of environmental variability by selection is increasing, and homogeneity is becoming important as a selection objective in animal breeding programs (Vandenplas *et al.* 2013; Rönnegård *et al.* 2013; Marjanovic *et al.* 2016), even with a low heritability and consequently hard to select. In addition, the homogeneity of a population directly affects animal welfare and profitability. Selection for homogeneity would result in more robust animals facing environmental challenges (Mormede and Terenina 2012). Homogeneity would also improve features related to robustness, such as survival, fertility and litter size (Formoso-Rafferty *et al.* 2016b), without decreasing the productivity and consequently increasing profitability (Formoso-Rafferty *et al.* 2018).

Understanding the forces that underlie the importance of the non-genetic component of phenotypic variance is a broad question in evolutionary biology (Hill and Mulder 2010). Classical selection methodology could be improved by managing the phenotypic variation that is partially under genetic control (Sorensen and Waagepetersen 2003). Several authors have focused on this problem and have estimated genetic parameters regarding residual variance in pigs (See 1998; Högberg and Rydhmer 2000; Damgaard *et al.* 2003), dairy cattle (Jaffrezic *et al.* 2000) and rabbits (Blasco *et al.* 2017).

Genetic correlation is explained by linkage, pleiotropy or a combination of these factors (Falconer and Mackay 1996). On the other hand, the skewness of the residual distribution provides information about the genetic correlation between the traits and their variability. Regarding the possible causes of the mean-variability genetic correlation, Sorensen and Waagepetersen (2003) demonstrated that the skewness of the distribution of the residuals determines the sign of the genetic correlation given that points in the skewed tail are more variable when they are located farther from the mean. Gutiérrez *et al.* (2006) observed this effect in litter traits in mice. Yang *et al.* (2011) demonstrated how a Box-Cox transformation (Box and Cox 1964) facilitates working with a non-skewed variable. Previous studies demonstrated that the mean-variability genetic correlation for litter size trait changed from -0.73 to 0.28 in rabbits and from -0.64 to 0.70 in pigs when the variable was transformed. However, the transformed variable is difficult to interpret (Pun *et al.* 2013). Although the skewness of the distributions can explain, to an extent, this genetic correlation, its skewness is not so often present in traits to provide significant and relevant genetic correlations. Therefore, there must be other reasons that make it appear in the data. The magnitude and sign of the genetic correlation between trait mean and its environmental variability is a concern given the correlated genetic response on one of them when selecting for the other. A wide range of genetic correlation estimates ranging from -0.93 to 0.97 has been reported in the literature (Hill and Mulder 2010; Formoso-Rafferty 2017).

Formoso-Rafferty *et al.* (2016a) carried out a successful divergent selection experiment for birth weight environmental variability in mice. The birth weight trait was normally distributed (Formoso-Rafferty *et al.*, 2016a), and a positive but low genetic correlation between birth weight and its environmental variability was observed. The statistical scale effect can be defined as the relationship between the mean and the variability of a trait in the sense that the higher the mean of a variable, the higher its variability. The estimated genetic correlation found by Formoso-Rafferty *et al.* (2016a) could be attributed to the statistical scale effect, which was suggested as a possible cause affecting the correlated trend in variability in another experiment to select weight gain in mice (Moreno *et al.* 2012).

The scale effect represents a potential cause for the observed correlation between a trait mean and its variance. The scale effect is caused by a direct relationship between the mean and variance, for instance when a trait has a constant coefficient of variation (CV) such that an increase in the mean also increases the variability. For most distributions the variance is directly connected to the mean, where the normal distribution is an important exception. In a Poisson distribution the variance is equal to the mean and in a Gamma distribution there is a direct relationship between the mean and CV (Rönnegård and Valdar 2012).

The scale effect is then a consequence of a manmade way to measure heterogeneity which is a difficult way to express the real natural determining process (Sun *et al.*, 2013). Among the implications of this mean-variability relationship are the concerns found in studies on Genome-Wide Association Study in which “gene by gene” and “gene by environment” interactions are confounded with marker effects on variability. Several methodologies have been developed to correct the effect (Rönnegård and Valdar 2012) and particularly the use of CV (Mackay and Lyman 2005). This and other transformations like monotonic (Sun *et al.*, 2013) or Box-Cox (Yang *et al.*, 2011) transformations were also essayed, but because of the ongoing debate on their feasibility it is advised to avoid these transformations (Shen and Rönnegård 2013).

The presence of an additive genetic variance for a trait in a population would imply the possibility of changing the mean by selection of that trait which would automatically generate the appearance of an additive genetic variance for the variability originated by the scale effect.

The CV is a statistical parameter defined as the ratio of the standard deviation (σ) to the mean (µ) to quantify the variability in a dimensionless manner. Thus, for a given CV, the higher mean, the higher standard deviation. Considering the CV to be fixed allows simplifying the scope of the study, as there is no natural direct relationship between mean and variability in a normally distributed variable. For a fixed CV, modifying the mean would automatically increase the standard deviation and vice versa. Since it is not common to have a CV constant across the whole range of a trait, a scenario in which the scale effect should be modulated was considered. Houle (1992) reported that the value of CV for measuring variability depends on the degree to which they correct for the relationships that exist between mean and variances. Consequently, modifying the mean by selection would not automatically change the variability of the trait to the same extent. Subsequently this would reduce the strength of the scale effect accordingly and, therefore, the additive genetic variance generated for the residual variance due to the scale effect.

The objective of this study was to quantify by simulation up to what extent the genetic correlation between a trait and its environmental variability could be attributed to the scale effect. The reduction of the scale effect due to an incomplete relationship between mean and variability, understood as the strength of the scale effect, were also assessed in the resulting additive genetic variance, thus affecting the residual variance.

## Materials and methods

The heteroscedastic model (HE) proposed by SanCristobal-Gaudy *et al.* (1998) was assumed to derive the additive genetic value *v_i_* affecting the residual variability, which has a Gaussian distribution, *i.e.*, $v_{i}∼N\left(0,\sigma_{v}^{2}\right)$. Under this model, the residual variance is heterogeneous and partially under genetic control. The simplest HE model was used:

$$y_{i}=\mu +u_{i}+{\text{e}}^{\frac{1}{2}\left(\eta +v_{i}\right)}\varepsilon_{i}$$

where *y_i_* is the record *i*, *μ* is the mean of the trait, *u_i_* the additive genetic effect, e*^η^* is the residual variance ($\sigma_{e}^{2}$) in the model HO, and *ε_i_* is a non-scaled residual with a Gaussian distribution of $\varepsilon_{i}∼N\left(0,1\right)$. It is assumed in this model that the corresponding vectors of additive genetic effects **u** and **v** can be correlated as follows:

$$\left(\begin{array}{c} u\\ v \end{array}\right)∼N\left(\left(\begin{array}{c} 0\\ 0 \end{array}\right),\left(\begin{array}{cc} \sigma_{u}^{2} & \rho \sigma_{u}\sigma_{v}\\ \rho \sigma_{u}\sigma_{v} & \sigma_{v}^{2} \end{array}\right)⊗A\right)$$

where **A** is the additive genetic relationship matrix, *ρ* is the genetic correlation and ⊗ is the Kronecker product. Note that the average value of *v* does not correspond with the mean residual variance due to the exponential nature of the model (Mulder *et al.* 2007).

The scale effect shows the relationship between the mean and the variability so that the higher the mean of a variable, the higher its variability. Thus, for instance, the standard deviation of a variable multiplied by a constant *k* is in turn multiplied by *k*, with its CV unaltered. This relationship between the level of a trait and its variability was assumed here to be for the performance level of each animal. Two simulation analyses were performed to explore the influence the scale effect can have on the genetic correlation between a trait and its residual variability. The first analysis was performed to compute the genetic correlation between a trait and the variability that occurs as a consequence of the scale effect. The second analysis was performed to assess the strength of the scale effect originating from the incomplete determination that the CV would have on the modulation of the magnitude of the additive genetic variance generated for the variability based on the scale effect.

### (i) Mean-variability genetic correlation generated by the scale effect

A total of 100 scenarios were simulated for the values of CV and *h^2^* ranging from 0.05 to 0.50 at 0.05 increments. Single records were simulated for a trait with a mean value (*µ*) of 100. The values for *µ*, CV and *h^2^* were fixed, but all the other parameters were derived from them. But for the sake of simplicity, residual and additive genetic effects were assumed to be the only random effects in the model. Simulations were performed in such a manner that higher phenotypes would have higher variance keeping the CV constant as expected by the scale effect. Phenotypic variance ($\sigma_{p}^{2}$), residual variance ($\sigma_{e}^{2}$) and additive genetic variance ($\sigma_{u}^{2}$) were initially defined as follows:

$$\begin{array}{l} \sigma_{p}^{2}={\left(CV*\mu \right)}^{2}\\ \sigma_{e}^{2}=\left(1-h^{2}\right)*\sigma_{p}^{2}\\ \sigma_{u}^{2}=h^{2}*\sigma_{p}^{2} \end{array}$$

These reference variances were initially considered to be homogeneous to simulate the trait level for each individual, and then they were considered heterogeneous and modulated by the trait level as a direct consequence of the scale effect. The simulations were performed in three steps:

1. First, each record *y_i_* of an animal *i* was simulated assuming a classical homoscedastic model (HO):
  $$y_{i}=\mu +a_{i}+e_{i}$$

where *a_i_* is the additive genetic effect and *e_i_* the residual effect that were randomly obtained from the following Gaussian distributions with unique variances:

$$\begin{array}{l} a_{i}∼N\left(0,\sigma_{u}^{2}\right)\\ e_{i}∼N\left(0,\sigma_{e}^{2}\right) \end{array}$$

1. Second, the simulated record *y_i_* described the level of the phenotype of the individual *i*, and the equivalent residual standard deviation in this individual ($\sigma_{e_{i}}$) with CV and *h^2^* remaining constant. The scaled phenotypic standard deviation was no longer unique and dependant on the magnitude of *y_i_* describing the performance level:
  $$\sigma_{p_{i}}=\left(CV*y_{i}\right)$$

The phenotypic standard deviation was, therefore, considered heterogeneous thus transferring this heterogeneity proportionally to both residual and additive genetic standard deviations that also became heterogeneous. The scaled residual standard deviation was then derived from the scaled phenotypic standard deviation and the heritability:

$$\sigma_{e_{i}}=\sigma_{p_{i}}*\sqrt{1-h^{2}}$$

The additive genetic variance also became heterogeneous in the simulation and also dependant on the scale effect. This is contrary to the definition of the HE model above, but necessary to avoid obtaining some possible residual variances higher than the phenotypic variance of the HO model used as reference. Therefore, the additive genetic standard deviation for the individual *i* ($\sigma_{u_{i}}$) also became scaled:

$$\sigma_{u_{i}}=\left(CV*y_{i}\right)*\sqrt{h^{2}}$$

Some minor negative phenotypes can accidentally be simulated for high CV values, resulting in negative values for $\sigma_{e_{i}}$. When this occurred, $\sigma_{e_{i}}$ was changed to a positive value. This happened for CV values higher than 0.25 with a maximum of 2.3% of the records in some replicates for CV = 0.5. This was empirically checked to confirm the effect on the genetic correlation was negligible.

1. Based on the HE model equation described above, the additive genetic value of individual *i* affecting variability (*v_i_*), would proportionally modify the residual variance in model HO ($\sigma_{e}^{2}$), which is used as a reference model, could lead to σei2σe2. Using an exponential model, the corresponding environmental additive genetic value of the individual *i* (*v_i_*) was then obtained from the following equation:
  $$v_{i}=2*ln\left(\frac{\sigma_{e_{i}}}{\sigma_{e}}\right)$$

To accommodate the additive genetic value affecting the trait, the *a_i_* simulated by the HO model was rescaled:

$$u_{i}=a_{i}\frac{\sigma u_{i}}{\sigma_{u}}$$

The genetic correlations between the additive genetic values for trait (*u*) and for the variability (*v*) were directly computed from the simulated values since they were available from the simulation. The mean genetic correlation of 10 independent replicates was computed within a scenario and 100,000 individuals were simulated for each scenario.

### (ii) Additive genetic variance of variability $\sigma_{v}^{2}$ generated by the scale effect strength

It is assumed that the simulation described above has the scale effect proportional to the value of the trait, but this assumption could be unrealistic. A direct determination of the variability from the level of the trait and the CV would then seem to be unrealistic, leading in addition to unreliable values for $\sigma_{v}^{2}$, particularly when the CV was higher than 0.25 (Hill and Mulder 2010). This second analysis was performed to study the strength of the scale effect due to an incomplete determination of *v* from the scale effect by defining a new parameter *r* (0 < *r ≤ 1*). This new parameter *r* would weaken the scale effect if it is less than 1; as a consequence the scale effect strength reduces the absolute value of the environmental additive genetic effect of the individual *i* as follows:

$$v_{i}=2*r*\ln\left(\frac{\sigma_{e_{i}}}{\sigma_{e}}\right)$$

Neither the value of the heritability (*h^2^*) nor the value of *r* will affect the genetic correlation between mean and residual variance, but the additive genetic variance of the variability $\sigma_{v}^{2}$ will be reduced by *r^2^*. In addition, this variance is dependent on the magnitude of the CV of the trait. This is a simple way to model the incomplete determination of the variability from the level of the trait, but many other models are possible.

Again, 10 replicates of 100,000 individuals were simulated per scenario. In this case, 200 scenarios were considered according to the values of CV and *r*. Here, CV ranged from 0.05 to 0.50, and *r* ranged from 0.05 to 1. Both variables were altered in increments of 0.05. Genetic coefficient of variation for environmental variance (GCV) is a measure of evolvability (Houle 1992). Therefore, for explanatory purposes, instead of presenting $\sigma_{v}^{2}$, the average of its square root value was computed across replicates within a scenario given that this value roughly represents the genetic coefficient of variation ($GCV\approx \sqrt{\sigma_{v}^{2}}$) of the variability (Hill and Mulder 2010).

The present work was motivated in the light of the correlated trend observed in the real scenario provided by the mice divergent selection experiment for birth weight environmental variance carried out by Formoso-Rafferty *et al.* (2016b). The information from this experiment was then used to discuss some aspects of the simulations regarding a real scenario. Estimated genetic parameters in this population were used as a reference to compare with simulations. In addition, the mean, standard deviation and CV were computed within five intervals of the data after the records were sorted for two different traits: birth weight and litter size. Based on this study design, the consistency of a unique CV value across the range of several variables will be discussed.

### Data availability

All simulations are available at https://gsajournals.figshare.com/s/30bbcbd31850ea2e18*cf*. Supplemental material available at FigShare: https://doi.org/10.25386/genetics.8862317.

## Results

**Figure 1:**
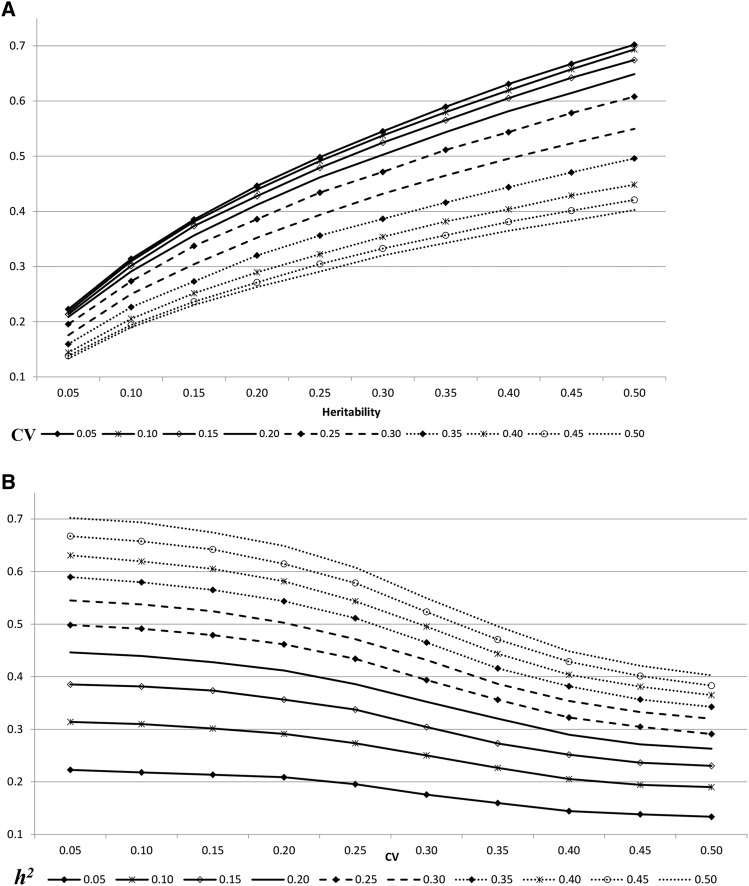
Evolution of mean-variability genetic correlation generated by the scale effect, across heritability (a) and across coefficient of variation (CV) (b).

The averages of the environmental variability genetic correlations within scenarios combining CV and heritability are reported in Table 1. The results ranged from 0.1335 to 0.7021. Standard errors of the means are not presented but ranged from 0.0009 to 0.0085. Figure 1 shows the genetic correlations obtained by the scale effect across heritability (Figure 1a) and across CV (Figure 1b). A growing trend of the genetic correlation across heritability was observed, and a decreasing trend was observed as the CV increased. The maximum value of genetic correlation was attained when heritability was maximal (0.50) and CV was minimal (0.05), and the minimum value resulted when heritabilities were minimal (0.05) and CV was maximal (0.50) (Table 1). Values obtained for CV higher than 0.25 might be slightly biased by the anomalous appearance of some minor negative residual variances, while the other scenarios were not affected by this artifact, when approaching the lines themselves for high CV values (Figure 1a). In a similar way, the negative trend of the genetic correlations across the CV became smoother after this CV threshold of 0.25 (Figure 1b).

**Table 1:** Mean environmental variability genetic correlation (*ρ*) derived from scenarios combining coefficient of variation (CV) and heritability (*h^2^*)

CV	*h^2^*									
	0.05	0.10	0.15	0.20	0.25	0.30	0.35	0.40	0.45	0.50
0.05	0.2227	0.3140	0.3853	0.4462	0.4983	0.5451	0.5895	0.6309	0.6674	0.7021
0.10	0.2179	0.3098	0.3814	0.4395	0.4913	0.5376	0.5797	0.6194	0.6576	0.6935
0.15	0.2135	0.3014	0.3736	0.4276	0.4791	0.5245	0.5651	0.6052	0.6421	0.6743
0.20	0.2087	0.2912	0.3564	0.4118	0.4616	0.5026	0.5436	0.5816	0.6146	0.6488
0.25	0.1955	0.2734	0.3375	0.3858	0.4340	0.4715	0.5114	0.5437	0.5783	0.6079
0.30	0.1756	0.2502	0.3042	0.3523	0.3937	0.4318	0.4651	0.4954	0.5233	0.5494
0.35	0.1596	0.2265	0.2730	0.3202	0.3562	0.3863	0.4160	0.4438	0.4707	0.4960
0.40	0.1442	0.2053	0.2516	0.2896	0.3224	0.3538	0.3818	0.4038	0.4286	0.4483
0.45	0.1381	0.1942	0.2364	0.2712	0.3046	0.3327	0.3566	0.3811	0.4013	0.4207
0.50	0.1335	0.1899	0.2304	0.2629	0.2909	0.3201	0.3426	0.3649	0.3830	0.4025

The mean GCV values obtained from 10 replicates under each of the different simulated CV values considering all the ranges of scale effect strength, are presented in Table 2. They increased linearly across the values considered for the strength of the scale effect defined by *r*, with growing slopes according to the growing CV. The values ranged from 0.0050 to 1.4854. Standard errors are not shown but also increased as simulated values of CV and *r* increased. The maximum was 0.0305 for CV = 0.50 and *r* = 1. Some of the GCV values obtained were higher than 0.69 and were inconsistent with the parameters reported in the literature.

**Table 2:** Genetic coefficient of variation (GCV) observed in simulated scenarios with different scale effect strength (*r*) and coefficient of variation (CV) values

*r*	CV									
	0.05	0.10	0.15	0.20	0.25	0.30	0.35	0.40	0.45	0.50
0.05	0.0050	0.0101	0.0154	0.0212	0.0278	0.0359	0.0460	0.0563	0.0660	0.0742
0.10	0.0100	0.0203	0.0309	0.0423	0.0555	0.0724	0.0916	0.1135	0.1329	0.1483
0.15	0.0150	0.0304	0.0463	0.0637	0.0835	0.1078	0.1382	0.1685	0.1980	0.2244
0.20	0.0201	0.0405	0.0619	0.0849	0.1113	0.1447	0.1842	0.2253	0.2645	0.2985
0.25	0.0250	0.0507	0.0775	0.1059	0.1385	0.1803	0.2320	0.2838	0.3319	0.3709
0.30	0.0301	0.0607	0.0925	0.1273	0.1671	0.2167	0.2764	0.3407	0.3973	0.4469
0.35	0.0351	0.0709	0.1085	0.1483	0.1946	0.2521	0.3222	0.3952	0.4612	0.5193
0.40	0.0401	0.0810	0.1239	0.1695	0.2222	0.2899	0.3704	0.4513	0.5313	0.5932
0.45	0.0451	0.0909	0.1392	0.1906	0.2509	0.3254	0.4161	0.5069	0.5983	0.6708
0.50	0.0501	0.1014	0.1551	0.2119	0.2787	0.3606	0.4628	0.5646	0.6606	0.7476
0.55	0.0552	0.1113	0.1701	0.2330	0.3050	0.3981	0.5073	0.6214	0.7284	0.8202
0.60	0.0604	0.1215	0.1857	0.2548	0.3328	0.4342	0.5505	0.6739	0.7916	0.8928
0.65	0.0652	0.1316	0.2008	0.2752	0.3605	0.4676	0.6002	0.7306	0.8600	0.9708
0.70	0.0701	0.1421	0.2161	0.2969	0.3896	0.5112	0.6432	0.7952	0.9292	1.0441
0.75	0.0753	0.1524	0.2321	0.3189	0.4165	0.5410	0.6874	0.8434	0.9997	1.1195
0.80	0.0804	0.1620	0.2478	0.3387	0.4449	0.5822	0.7416	0.9073	1.0596	1.1938
0.85	0.0852	0.1721	0.2623	0.3601	0.4731	0.6126	0.7842	0.9646	1.1220	1.2695
0.90	0.0902	0.1824	0.2784	0.3812	0.5006	0.6510	0.8373	1.0194	1.1918	1.3382
0.95	0.0951	0.1924	0.2936	0.4048	0.5265	0.6887	0.8775	1.0813	1.2549	1.4250
1.00	0.1004	0.2024	0.3096	0.4228	0.5560	0.7248	0.9188	1.1280	1.3232	1.4854

Values greater than 0.69 are considered meaningless and are shaded.

## Discussion

The results show that the scale effect can partly justify at least the positive genetic correlations estimated between the mean and the environmental variability for some traits in some populations. As a consequence, modifying the mean of a trait by selection can bring about a change in the variability, as well as the reverse, modifying the variability might imply a modification of the mean in the same direction as a consequence of the scale effect. The genetic correlation generated by the scale effect assuming a constant CV would range from 0.13 to 0.70 depending on the heritability and CV in a range from 0.05 to 0.50 for both parameters. The additive genetic variance for the environmental variability, due to the scale effect, could be considered as an incomplete determination thus reducing its strength.

The genetic correlation between the mean and the variability is a matter for concern given that the environmental variability can be modified by correlated selection (Damgaard *et al.* 2003; Huby *et al.* 2003). In fact, correlated responses exhibit variability when selecting to increase the mean (Moreno *et al.* 2012), and changes in the mean trait are also present when selecting for variability (Formoso-Rafferty *et al.* 2016a). Hill and Mulder (2010) reviewed estimations of genetic correlations, reporting any value within the possible range. A more recent review suggests that this parameter tends to be more frequently positive, *i.e.*, birth weight, 0.42 and 0.44 in cattle (Neves *et al.* 2011; Fina *et al.* 2013), 0.55 and 0.62 in pigs (Sell-Kubiak *et al.* 2015b), also positive for adult weight, 0.30 and 0.79 in rainbow trout (Sae-Lim *et al.* 2015) and 0.58 in tilapia (Marjanovic *et al.* 2016); milk yield, 0.60 in dairy cattle (Rönnegård *et al.* 2013); teat count, 0.80 in pigs (Felleki and Lundeheim 2015); litter size, 0.49 in pigs (Sell-Kubiak *et al.* 2015a). Lower positive values were observed for other traits such as morphology traits in tilapia (0.11-0.37 (Marjanovic *et al.* 2016) or 0.06 for conformation scores in cattle (Neves *et al.* 2011). Other traits exhibited different signs and magnitudes, ranging from -0.06 to 0.43 for egg color (Mulder *et al.* 2016). Alternatively, lower magnitude values for weight gain were observed in different periods: 0.17, 0.02 and -0.09 (Neves *et al.* 2011). Finally, negative values were also identified, such as -0.52 for litter size in pigs (Felleki *et al.* 2012), -0.23 to -0.45 for chicken birth (Mulder *et al.* 2009; Wolc *et al.* 2009) and -0.16 for adult body weight in rainbow trout (Janhunen *et al.* 2012). The sign and magnitude of any genetic correlation was classically explained by linkage, pleiotropy or a combination of both (Falconer and Mackay 1996). Regarding the relationship between the mean level and the variability, a type of pleiotropic correlation would occur from the skewed distribution of the trait (Yang *et al.* 2011; Sorensen and Waagepetersen 2003; Ros *et al.* 2004; Gutiérrez *et al.* 2006). From this brief review, there is clearly an increased frequency of positive correlations between the mean of a trait and its variability suggesting that the scale effect could be generating these effects, but the effects seem to be different for different type of traits. For example, individual weight traits or dairy traits seem to be related to positive correlations while negative values appear more frequently in reproductive traits. Some populations with an important selection success would have had those animals with a prospect of low performance culled. In litter size, for instance, with repeated measures, some animals with low performance records would affect the mean litter size and these animals would not be selected, thus reducing the variability while increasing the mean value. Therefore, the final genetic correlation in each scenario would depend on the population status concerning selection, the trait and the influence of other causes affecting genetic correlation, but the scale effect seems to be present in many of them. In addition, assumptions established in these simulations, even when looking realistic, are only theoretical and avoid complexity. In fact the closer evolution lines of Figure 1a suggest that high values of variability are not easily supported by nature.

This simulation study revealed that the genetic correlation generated by the scale effect would range from 0.15 to 0.70 depending on the heritability and CV values of the trait in a particular population. The mouse experiment motivating this research had an undesired correlated genetic response for variability that appeared in the mean birth weight when selecting for environmental variability (Formoso-Rafferty *et al.* 2016a). An updated estimation of the genetic parameters in this population after 18 generations of selection under the reported methodology provided a genetic correlation of 0.3169 with a global heritability of 0.1329 (Formoso-Rafferty *et al.* 2017), and the trait had a computed CV of 0.1470. According to the values reported in Table 1, the expected genetic correlation generated by the scale effect for a heritability of 0.15 and a CV of 0.15 was 0.3736. Thus, the scale effect would explain the estimated genetic correlation in this case in which the relationship between mean and variability is assumed to be a constant as it directly depends on CV. In general, for a weight type trait with heritability between 0.25 and 0.35 and a CV between 0.15 and 0.25, the genetic correlation generated by the scale effect would range from 0.43 to 0.57. Instead, a litter size type trait, with heritability under 0.10 and CV between 0.10 and 0.15, the expected genetic correlation generated by this effect would be between 0.21 and 0.31. The lowest expected genetic correlations generated by this effect would be defined for low heritability traits with high CV, and the highest would be inversely high heritabilities with low CV.

**Figure 2:**
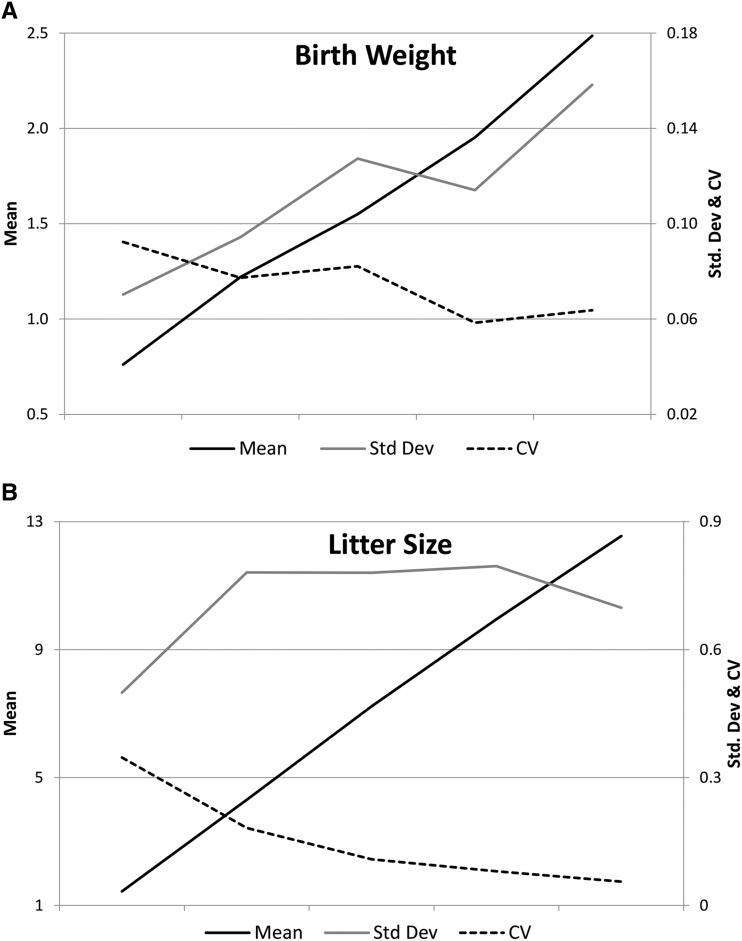
Mean, standard deviation (Std. Dev) and Coefficient of Variation (CV) of a) birth weight and b) litter size across five equal size sorted groups.

The simulation performed in this research to define the phenotypic standard deviation as directly proportional to the mean and CV lead to the appearance of an additive genetic variance affecting the environmental variability. Using the square root as an approach for GCV, a simulated scenario of *h^2^* and CV equal to 0.15 resulted in a GCV of 0.3094, but the estimated value by Formoso-Rafferty *et al.* (2016a) updated to the 18^th^ generation was 0.1929, which is significantly different to that obtained by simulation. This indicated that there was a lower sensitivity, suggesting that the assumed proportionality between mean and standard deviation would be incomplete. For example, Figure 2 presents the evolution of the mean, standard deviation and CV of birth weight (Figure 2a), and litter size (Figure 2b) across five sorted groups with the same number of records ordered by the trait value in this experiment. A visual scale effect can be observed for birth weight with increased standard deviation accompanying the increase in the mean value. However, the evolution of the standard deviation is not proportional to the mean, leading to a reduction in the CV. The pattern of the trend for the case of the litter size is different. The scale effect has a greater effect for low values of the trait with an increase in the standard deviation. The effect stabilizes for intermediate values and decreases for high values of litter size. The scale effect would be surpassed by other causes of genetic correlation between the trait and variability; thus, for highly selected populations for litter size in prolific species, a negative genetic correlation would exist between this trait and its environmental variability as reported by Sorensen and Waagepetersen (2003) and Felleki *et al.* (2012) in pigs and Ibáñez-Escriche *et al.* (2008) in rabbits. Part of these genetic correlations can be attributed to the skewness of the distribution of residuals. Yang *et al.* (2011) estimated negative genetic correlations in untransformed litter size in pigs and rabbits that became positive after a Box-Cox transformation. However, the genetic correlation would remain positive in the previously mentioned mice population (Formoso-Rafferty *et al.* 2016a).

To account for the impact of the scale effect, the second simulation was performed to demonstrate different strengths of the scale effect (*r*). Different positive *r* values were employed to determine how GCV would be affected. For the sake of simplicity, a constant value for the entire range of the trait was assumed although the value of *r* can differ for different values of the interval (Figure 2a) or even be asymmetric. Thus, it is possible for some values of the trait to be positive, whereas others are negative (Figure 2b).

Table 2 presents the GCV observed for the simulated scenarios with different scale effect strength (*r*) and CV values. The CV values ranged from 0.0050 (*r* = 0.05 and CV = 0.05), increasing with the value of the simulated parameters up to 1.4854 (*r* = 1.00 and CV = 0.50). Compared with the values reviewed by Hill and Mulder (2010), the estimations of the posterior revision reported above (Neves *et al.* 2011; Felleki *et al.* 2012; Janhunen *et al.* 2012; Rönnegård *et al.* 2013; Fina *et al.* 2013; Sae-Lim *et al.* 2015; Felleki and Lundeheim 2015; Sell-Kubiak *et al.* 2015a; Sell-Kubiak *et al.* 2015b; Mulder *et al.* 2016; Marjanovic *et al.* 2016) and with the exception of the anomalous unreliable estimation for birth weight in mice by Gutiérrez *et al.* (2006) as justified by Pun *et al.* (2013), GCV values greater than 0.69 were never reported and might even be considered meaningless (Hill and Mulder 2010). High-scale effect strength is not typical and would never be possible in the context of high CV values. The value of 0.1929 was estimated for GCV in the mice experiment (Formoso-Rafferty *et al.* 2016a) based on a CV value of 0.1470; these values roughly approach 0.1857 (*r* = 0.60) and 0.2008 (*r* = 0.65) from Table 2 for a CV = 0.15. This strength would be reasonable in the light of the trends of the standard deviation and CV presented in Figure 2a. Then, the correlated response in the mice selection experiment can be completely explained by the scale effect modulated in this case by the strength 0.60 of the scale effect. According to the GCV values in the literature referred above, common values of GCV are in the range between 0.15 and 0.50. Assuming a CV less than 0.25, the strength of the scale effect is expected to be any point of the space defined for the parameter between 0 and 1.

To conclude, this simulation study demonstrates how the scale effect can justify the genetic correlation that often appears between the mean and the environmental variability of some traits in some populations, and the additive genetic variance for the variability that might simply be generated by this scale effect. Breeders should be well aware of this fact when their selection objective is to increase the mean of a trait jointly with the reduction of its variability to develop an adequate selection index for this trait or to find an adequate transformation of the trait to remove the scale effect. The issue is also important in the search of vQTL in which it would help to make the mean and variable independent (Rönnegård and Valdar 2012). Further studies are needed in each case given that the scale effect could vary across the range of traits and even counterbalanced by other factors affecting the genetic correlation.
